# Survival of Adults with Acute Lymphoblastic Leukemia in Germany and the United States

**DOI:** 10.1371/journal.pone.0085554

**Published:** 2014-01-27

**Authors:** Dianne Pulte, Lina Jansen, Adam Gondos, Alexander Katalinic, Benjamin Barnes, Meike Ressing, Bernd Holleczek, Andrea Eberle, Hermann Brenner, the GEKID Cancer Survival Working Group

**Affiliations:** 1 Division of Clinical Epidemiology and Aging Research, German Cancer Research Center (DKFZ), Heidelberg, Germany; 2 Cardeza Foundation and Division of Hematology, Department of Medicine, Thomas Jefferson University, Philadelphia, Pennsylvania, United States of America; 3 Institute of Cancer Epidemiology, University of Lübeck, Lübeck, Germany; 4 National Center for Cancer Registry Data, Robert Koch Institute, Berlin, Germany; 5 Cancer Registry of Rhineland-Palatinate, Institute of Medical Biostatistics, Epidemiology, and Informatics, Johannes Gutenberg University Mainz, Mainz, Germany; 6 Saarland Cancer Registry, Saarbrücken, Germany; 7 Cancer Registry of Bremen, Leibniz Institute for Prevention Research and Epidemiology – BIPS, Bremen, Germany; 8 German Cancer Consortium (DKTK), Heidelberg, Germany; Utrecht University, Netherlands

## Abstract

**Background:**

Adulthood acute lymphoblastic leukemia (ALL) is a rare disease. In contrast to childhood ALL, survival for adults with ALL is poor. Recently, new protocols, including use of pediatric protocols in young adults, have improved survival in clinical trials. Here, we examine population level survival in Germany and the United States (US) to gain insight into the extent to which changes in clinical trials have translated into better survival on the population level.

**Methods:**

Data were extracted from the Surveillance, Epidemiology, and End Results database in the US and 11 cancer registries in Germany. Patients age 15–69 diagnosed with ALL were included. Period analysis was used to estimate 5-year relative survival (RS).

**Results:**

Overall 5-year RS was estimated at 43.4% for Germany and 35.5% for the US (p = 0.004), with a decrease in survival with increasing age. Survival was higher in Germany than the US for men (43.6% versus 37.7%, p = 0.002) but not for women (42.4% versus 40.3%, p>0.1). Five-year RS estimates increased in Germany and the US between 2002 and 2006 by 11.8 and 7.3 percent units, respectively (p = 0.02 and 0.04, respectively).

**Conclusions:**

Survival for adults with ALL continues to be low compared with that for children, but a substantial increase in 5-year survival estimates was seen from 2002 to 2006 in both Germany and the US. The reasons for the survival differences between both countries require clarification.

## Introduction

Acute lymphoblastic leukemia (ALL) is a rare cancer, especially in adults. Survival estimates for patients with ALL are high for children, both in clinical trials [1] and population based studies [2], but decrease rapidly with age [3], [4], and adult ALL is frequently fatal [5], [6]. Aggressive treatment of ALL has demonstrated increased survival in young and middle aged adult patients in clinical trials [5]. However, these changes have not yet been confirmed on the population level.

Because of the rarity of ALL in adults, there are relatively few population level data available concerning survival of patients with ALL and most available data come from the Surveillance, Epidemiology, and End Results (SEER) database in the United States (US) [2]–[4], [6], [7] and cancer registries from Nordic countries [8], [9]. In the past, estimates of population level survival for patients with leukemia in Germany have been limited to aggregate data for all forms of leukemia [10] due to lack of a unified, high quality population level database. Recently, a collaborative effort between the German Cancer Research Center and population based cancer registries in Germany covering 11 federal states has allowed for evaluation of population level survival for rare cancers in Germany, including evaluation of age and sex specific survival [11].

Here, we examine survival of adults diagnosed with ALL in Germany by age and gender and compare survival to that seen in patients with ALL in the US.

## Methods

### Data Sources

A detailed description of the cancer registries from which data were obtained has been published previously [11]. Briefly, data extracted from cancer registries throughout Germany covering 11 federal states, representing a total base population of 33 million people, were included (Table 1). Patients age 15 or older with a primary diagnosis of ALL (ICD-10 code C91.0) in 1997–2006 and with mortality follow up through December, 2006 were included. Cancer topography, morphology, and behavior were originally coded in accordance with the International Classification of Disease for Oncology (ICD-O)-3 guidelines and later converted into ICD-10 using ‘IARCcrgTools’ [12]. Patients with both B-cell and T-cell acute lymphoblastic leukemia are covered under this diagnostic code in ICD-10. For some registries, data were available starting from later years only. Cases both with and without preceding cancers were included. Because there were data quality issues for patients age 70+ in some of the German registries, only data for patients age 15–69 were included. In order to compare population level survival for ALL in Germany with survival in the United States (US), data from the Surveillance, Epidemiology, and End Results (SEER13) database were analyzed [13]. The same inclusion criteria as for patients from the German cancer registries were applied for the same time period. The SEER13 database includes data from 13 regional cancer centers in the US, covering a population of about 39 million people. Centers are chosen for inclusion based on their high quality and epidemiologically interesting population groups. The SEER population is considered to be similar to the general US population with respect to most sociodemographic characteristics [13], although it may be more affluent than average and may have slightly higher than average survival for some cancers [14].

**Table 1:** Patients diagnosed with acute lymphoblastic leukemia at age 15–69 included in this analysis.

Registry	Population base (million)	Years Included	Cases registered	% DCO (excluded)	Cases in the analysis	Median age at diagnosis	% Microscopically confirmed
**Bavaria** a	8.13	2002–06	205	8.3	188	38.0	100.0
**Brandenburg**	2.55	1997–2006	175	11.4	155	37.0	100.0
**Bremen**	0.66	1998–2006	34	5.9	32	36.5	96.9
**Hamburg**	1.75	1997–2006	111	4.5	106	36.5	100.0
**Mecklenburg-Vorpommern**	1.69	1997–2006	115	12.2	101	38.0	100.0
**Lower Saxony**	7.98	2001–06	185	10.3	166	40.0	97.5
**North Rhine-Westphalia** a	2.62	1997–2004	94	7.4	87	39.0	98.8
**Rhineland-Palatinate** a	0.52	1998–2006	25	24.0	19	36.0	89.5
**Saarland**	1.04	1997–2006	67	1.5	66	42.0	98.5
**Saxony**	4.25	1997–2006	215	7.4	199	40.0	98.5
**Schleswig-Holstein** a	1.85	1999–2006	69	18.8	56	38.5	100.0
**Total** **SEER**	33.0439	1997–2006	12952314	9.30.3	11752307	39.037.0	99.099.3

DCO = death certificate only.

a Selected administrative districts only.

11 German registries, 1997–2006.

### Ethics

The data contained in the databases under study is stripped of all sensitive identifying information prior to being made available to researchers. Thus, no additional specific informed consent was required for analysis of the anonymised data in this project. Written consent was neither possible nor desirable as it would represent a link to individual patients and thus constitute a risk of disclosure that would not otherwise exist.

### Statistical Methods

Five-year relative survival estimates for the time period 2002–06 were calculated using period analysis [15]. Period analysis, first introduced in 1996 [16], provides more up-to-date survival estimates than traditional cohort based analysis. This is achieved by “left truncation” of all observations at the beginning of the period of interest (in our case: the beginning of 2002). In particular, it has been shown by empirical evaluation, that period estimates of 5-year relative survival for a given period quite closely predict 5-year relative survival later observed for patients diagnosed during the period of interest [16]–[18]. Age-adjusted survival estimates were derived by computing weighted sums of age-specific survival estimates using weights according to the proportion of cases in various age groups (15–24, 25–39, 40–59, and 60–69) in Germany.

Age intervals were chosen based on frequency of ALL at various ages and for potentially clinically significant age breaks, i.e. patients over age 60 are much less likely to be eligible for a traditional hematopoietic stem cell transplant, which may affect survival. In addition, in the US, universal health insurance is available only to patients age 65 and older (Medicare) and thus older patients may have a relative survival advantage at that age compared to younger patients who may be uninsured. Because survival in ALL varies with age and gender, we examined survival by major age groups and by gender. Differences in survival between men and women, as well as between patients in Germany and the US, were tested for statistical significance, overall and by single age groups, using model-based period analysis [19]. Additionally, model-based period analysis was employed to estimate most recent changes in 5-year relative survival within the 2002–2006 period.

Because the number of cases reported to cancer registries by death certificate only (DCO) in the German database was still high, the impact of the exclusion of DCO cases in the computation of the survival estimates was estimated by providing plausibility ranges for survival estimates. The plausibility range is derived by computing relative survival once after exclusion of DCO cases (upper limit of the estimate) and once by multiplying the relative survival estimate obtained after exclusion of DCO cases by one minus the percentage of DCO cases (lower limit). The latter estimate was suggested by Berrino et al. [20] to account for the overoptimistic estimate of relative survival after exclusion of DCO cases. Brenner and Holleczek [21] have shown that these two estimates provide a plausibility range for true survival, as the former estimate is expected to overestimate true survival, and the latter estimate is expected to underestimate true survival under plausible assumptions.

Relative survival was calculated as the ratio of actual survival to expected survival. Expected survival was estimated according to the Ederer II method [22] using national life tables stratified by age, sex, and calendar year obtained from German Federal Statistical Office. Relative survival estimates for the US patients were calculated using US sex, age, calendar year, and race specific life tables published by the Center for Disease Control (CDC) [23].

All calculations were carried out using SAS software (version 9.2), using macros developed for standard and modeled period analysis [19], [24].

## Results

Overall, 1295 patients age 15–69 were identified in the German database. After exclusion of DCO cases (9.3%), 1175 cases remained for analysis. Median age at diagnosis was 39 years (for patients age 15–69 at diagnosis), with some variability between registries, ranging from 36 in Rhineland-Palatinate to 42 years in Saarland (Table 1). The percentage of DCO cases varied between databases, ranging from 1.5% in Saarland to 24% in Rhineland-Palatinate.

Within the SEER database, 2314 patients were identified using the same criteria as above and after exclusion of 7 (0.3%) cases identified by DCO, 2307 remained for analysis. Median age at diagnosis for patients in the SEER database was 37 years for patients age 15–69 at time of diagnosis.

Overall age standardized five-year relative survival was 43.4% in Germany and 35.5% in the US (Table 2). There was a trend towards higher 5-year survival in Germany for each age group, which reached significance overall and for ages 40–59 at +7.9 percent units and +15.9 percent units, respectively. Plausibility ranges, which take potential overestimation of survival due to the higher proportions of DCO cases in Germany into account, suggest that the latter are unlikely to explain the higher survival estimates in Germany compared to the US as the lower ends of the plausibility range were still higher than the US estimates in each case.

**Table 2:** Five year relative survival of patients with acute lymphoblastic leukemia in Germany and the US in 2002–06, overall and by age.

		Germany					US			
Age	N	RS	SE	Plausibility range		N	RS	SE	Diff	P (Model)
**15–24**	322	59.2	3.8	56.9–59.2		680	54.9	2.8	+4.3	0.5232
**25–39**	280	47.7	4.2	43.0–47.7		580	42.3	3.0	+5.4	0.4491
**40–59**	331	40.0	3.9	35.7–40.0		759	24.1	2.3	+15.9	0.0041
**60–69**	242	21.8	4.5	18.9–21.8		288	17.7	3.5	+4.1	0.2725
**Overall** a	1175	43.4	2.0	39.8–43.4		2307	35.5	1.4	+7.9	0.0040

N = number of cases.

RS = 5-year relative survival.

SE = standard errors.

Diff = difference in survival between Germany and the United States.

a Age-standardized.

Interestingly, most of the difference in survival seems to be related to differences in survival for men. When survival was examined by gender and age, men had a higher five year relative survival in Germany (Table 3). The survival advantage of male patients from Germany was statistically significant overall and for ages 40–59 at +5.9 and +19.0 percent units, respectively. No statistically significant difference in survival was seen for women at any age and there was a trend towards lower survival in Germany for ages 15–39 (Table 3). It should be noted that there was a large difference in the point estimate of survival for women age 40–59 in Germany versus the US at +12.6 percent units, but the difference was not statistically significant, possibly due to the small number of cases. Survival decreased with age in both men and women. This finding was statistically significant for all populations except for women age 50–59 compared to 60–69.

**Table 3:** Five year relative survival of patients with acute lymphoblastic leukemia in Germany and the US in 2002–06, by gender and age.

Men									
		Germany				US			
Age	N	RS	SE	Plausibility range	N	RS	SE	Diff	P (Model)
**15–24**	214	59.2	4.7	57.4–59.2	460	51.8	3.4	+7.4	0.2767
**25–39**	188	50.0	5.0	45.4–50.0	357	37.8	3.8	+12.2	0.1702
**40–59**	197	42.3	5.2	37.3–42.3	392	23.3	3.1	+19.0	0.0027
**60–69**	114	17.4	5.8	14.4–17.4	154	14.3	4.4	+3.1	0.6117
**Overall^a^**	713	43.6	2.6	40.0–43.6	1363	32.7	1.8	+10.9	0.0021

Women									
		Germany				US			
Age	N	RS	SE	Plausibility range	N	RS	SE	Diff	P (Model)
**15–24**	108	59.5	6.2	56.3–59.5	220	61.7	4.9	−2.2	0.6143
**25–39**	92	43.9	7.6	39.2–43.9	223	49.2	4.8	−5.3	0.6124
**40–59**	134	37.2	5.7	33.7–37.2	367	24.6	3.5	+12.6	0.3101
**60–69**	128	25.1	6.8	22.8–25.1	134	23.0	5.6	+2.1	0.3854
**Overall^a^**	462	42.4	3.3	39.0–42.1	944	40.3	2.3	+2.1	0.4544

N = number of cases.

RS = 5-year relative survival.

SE = standard errors.

Diff = difference in survival between Germany and the United States.

a Age-standardized.

Because race may be used as a proxy for socioeconomic status and may convey risk of sub-optimal treatment in the US and there are known differences in survival by race for patients with acute leukemias in the US [6], we examined survival only for patients listed as “white” in the US as well. Full results are shown in table S1. Overall there was little change in the pattern seen when all patients were included, with continued better survival for patients in Germany, especially for men, but with larger confidence intervals and thus fewer differences that were statistically significant.

In order to examine recent changes in survival of patients with ALL, survival in 2002 and 2006 was compared in each country. In Germany, there was a significant improvement in survival between 2002 and 2006, by +11.8 (p = 0.02) percent units (Table 4).

**Table 4:** Trends in 5-year relative survival from 2002 to 2006 by sex in Germany.

	2002		2006		Difference	
Group	RS	SE	RS	SE	Diff	P (model)
**Overall**	36.9	3.4	48.7	2.9	11.8	0.0240
**Male**	31.9	4.1	52.8	3.7	20.9	0.0016
**Female**	44.0	5.6	41.5	4.5	−2.5	0.7620

RS = 5-year relative survival.

SE = standard errors.

Diff = difference in survival between 2002 and 2006.

In the US, there was a statistically significant increase, at +7.3 percent units (Table 5).

**Table 5:** Trends in 5-year relative survival from 2002 to 2006 by sex in the US.

	2002		2006		Difference	
Group	RS	SE	RS	SE	Diff	P (model)
**Overall**	31.3	2.2	38.6	1.9	7.3	0.0367
**Male**	27.5	2.7	37.2	2.6	9.7	0.0280
**Female**	37.9	3.7	40.8	3.0	2.9	0.5994

RS = 5-year relative survival.

SE = standard errors.

Diff = difference in survival between 2002 and 2006.

In both countries, the increase in survival was limited to male patients, at +20.9 percent units and +9.7 percent units in Germany and the US, respectively. Survival for women with ALL was virtually unchanged in either country between 2002 and 2006. In both countries, survival for men was lower than for women in 2002 but similar to or higher than for women in 2006.

## Discussion

Five year survival for young and middle aged adults with ALL was higher in Germany than in the US overall. There was a trend towards higher survival estimates for all ages, even though this trend only reached significance for the age group 40–59. Survival estimates were higher in Germany for men but not for women. Survival estimates were higher overall in 2006 than in 2002 in both countries, but the increase was restricted to men in each country. Survival decreased rapidly with age in both countries.

The reasons for the differences observed between Germany and the US are not obvious. Treatment guidelines, which recommend aggressive combination chemotherapy with or without hematopoietic stem cell transplantation for young and fit patients, are similar in both countries [25], [26]. The use of tyrosine kinase inhibitors is recommended for Philadelphia chromosome positive patients in each country. Additionally, there is no routine screening test available for ALL, making differences in the timing of diagnosis unlikely. It is possible that lack of health insurance, possibly leading to delays in treatment or sub-optimal treatment, may contribute to lower survival in the US, but the data do not show a large difference in survival at younger ages in the US as one might expect if lack of insurance were a major issue [27], [28].

Female gender is considered a good prognostic indicator for pediatric patients with ALL [29], [30]. However, these differences could be at least partly explained by differences in biological features of the ALL [30]. The literature is less clear with respect to the role of gender in survival of adults with ALL, with some studies showing better results for female patients, others finding better outcomes for male patients [31], [32]. In our study, men had much worse prognosis than women at the beginning of the period of investigation (2002) in both countries, but men seem to have caught up and even achieved higher survival than women in Germany in 2006, possibly reflecting greater benefit of newer therapies or new applications of existing therapies (i.e. the use of tyrosine kinase inhibitors in BCR_Abl positive leukemias or the use of pediatric protocols in young adults) to men than women. However, given the rarity of the condition, some amount of random fluctuation can not be ruled out.

Increasing age is generally considered a poor prognostic indicator in acute leukemia and we found that, as expected, survival decreased rapidly with increasing age in Germany and the US. Patients age 15–24 had a greater than 10 percent units better chance of 5-year survival than patients in the next older age group of 25–39. This may reflect use of more aggressive treatment in younger patients, higher probability of good prognosis leukemias in younger patients, or some combination of these two factors. Previous studies have found a higher prevalence of poor prognostic markers, including the Philadelphia chromosome, among older patients [31], [33] as well as a lower prevalence of good prognostic markers [34]. Additionally, patients over age 25 may not be offered aggressive therapy or may have increased mortality with aggressive therapy, leading to an overall decrease in survival [35]. Because of the relatively small number of cases available for analysis, even with the large databases examined, we were not able to determine whether the observed changes in survival during the period of investigation were distributed equally at all ages or if some age groups experienced greater or lesser change.

Strengths of this study include the use of large, population based databases to determine survival estimates and inclusion of data from a number of registries. This allows for detailed estimates of survival in a rare tumor such as ALL on the population level. Population level survival can vary greatly from survival observed in clinical trials [36], making this information important in determining the “real world” outcomes of patients with a given condition. Additionally, the use of the large databases allows for examination of sub-groups of patients, which may help identify areas of concern, i.e. patient populations for whom survival is not changing or is worsening, even as survival improves overall for a given condition. Finally, the use of period analysis and modelled period analysis provide the most up-to-date estimates of survival possible.

In considering our work, several limitations should be considered. First, despite the use of the large population based databases, the relative rarity of ALL makes it difficult to analyze survival patient subgroups with precision. For example, a number of relatively large differences in point estimates of survival were not statistically significant due to small numbers and resultant large confidence intervals. This makes the possibility of a type two error high. Second, the databases used for this analysis did not contain important information on therapy, such as chemotherapy, hematopoietic stem cell transplantation, or inclusion in clinical trials. Therefore, no conclusions can be drawn with respect to possible treatment differences between Germany and the US.

Third, in the absence of a national death index in Germany, most cancer registries rely on record linkage with vital statistics from the region that they cover and may miss deaths among patients who move out of the region. Number of patients lost to follow up is not directly assessed by most registries with the exception of two registries: Hamburg and Bremen. Nevertheless, previous validation studies have suggested potential overestimation of survival due to deaths missed by migration to be very small [11]. Specifically, the effect of migration was directly measured for two registries covering Hamburg and Bremen and patients who emigrated were censored at the date of emigration. If these patients were instead listed as alive, the rate of apparent survival was raised by less than 1% unit. The effect of migration on larger regional databases is expected to be even lower.

There is a theoretical concern about incomplete inclusion of patients given that some registries have 100% microscopically confirmed cases which might suggest incomplete registration of cases. However, elimination of the registries with 100% microscopically confirmed cases did not materially change the results (data not shown) and the percentage of microscopically confirmed cases in the SEER data is also quite high (99.3%), suggesting that any bias that might be present is present equally in each registry.

Finally, the higher proportion of DCO notifications in Germany might affect survival estimates. We aimed to address this concern by providing plausibility ranges for the relative survival estimates in Germany. As the lower end of the plausibility range by far exceeded the survival estimates for the US in most cases, it is unlikely that the major survival differences between both countries are due to differences in data quality and completeness of case ascertainment.

In summary, higher 5-year relative survival estimates were observed on the population level for adults with ALL in Germany compared to the US. Some increase in survival was seen in each country, but survival estimates did not reach those observed in childhood for either country. Survival decreased with age. A major survival disadvantage of male patients seems to have been overcome in the period of investigation during which a strong increase in survival was seen among male, but not among female patients.

## Supporting Information

Table S1 **Analysis of five year survival for patients in Germany and the US when only patients listed as “white” were included in the analysis in the US.**
(DOCX)
