# Supplementary material for: Survival of Adults with Acute Lymphoblastic Leukemia in Germany and the United States
Source: PLoS One. 2014 Jan 27;9(1):e85554. doi: 10.1371/journal.pone.0085554 (PMC3903479; doi:10.1371/journal.pone.0085554)
Supplement: Table S1 — Analysis of five year survival for patients in Germany and the US when only patients listed as “white” were included in the analysis in the US. (DOCX) [file pone.0085554.s001.docx]

1. Overall survival including all patients

|  |  | **Germany** |  |  |  |  | **US** |  |  |  |
| --- | --- | --- | --- | --- | --- | --- | --- | --- | --- | --- |
| **Age** | **N** | **RS** | **SE** | **Plausibility range** |  | **N** | **RS** | **SE** | **Diff** | **P (Model)** |
| **15-24** | 322 | 59.2 | 3.8 | 56.9-59.2 |  | 569 | 58.2 | 3.0 | +1 | 0.9564 |
| **25-39** | 280 | 47.7 | 4.2 | 43.0-47.7 |  | 465 | 42.0 | 3.3 | +4.4 | 0.3757 |
| **40-59** | 331 | 40.0 | 3.9 | 35.7-40.0 |  | 604 | 24.3 | 2.6 | +15.7 | 0.0111 |
| **60-69** | 242 | 21.8 | 4.5 | 18.9-21.8 |  | 239 | 17.6 | 3.9 | +4.2 | 0.2880 |
| **Overall^a^** | 1175 | 43.4 | 2.0 | 39.8-43.4 |  | 1877 | 36.4 | 1.6 | +7.0 | 0.0182 |

b) Men

|  |  | **Germany** |  |  |  |  | **US** |  |  |  |
| --- | --- | --- | --- | --- | --- | --- | --- | --- | --- | --- |
| **Age** | **N** | **RS** | **SE** | **Plausibility range** |  | **N** | **RS** | **SE** | **Diff** | **P (Model)** |
| **15-24** | 214 | 59.2 | 4.7 | 57.4-59.2 |  | 393 | 53.5 | 3.6 | +5.7 | 0.4558 |
| **25-39** | 188 | 50.0 | 5.0 | 45.4-50.0 |  | 284 | 38.6 | 4.2 | +11.4 | 0.1768 |
| **40-59** | 197 | 42.3 | 5.2 | 37.3-42.3 |  | 310 | 23.4 | 3.5 | +18.9 | 0.0060 |
| **60-69** | 114 | 17.4 | 5.8 | 14.4-17.4 |  | 131 | 16.4 | 4.9 | +1 | 0.8379 |
| **Overall^a^** | 713 | 43.6 | 2.6 | 40.0-43.6 |  | 1118 | 33.8 | 2.0 | +9.8 | 0.0105 |

c) Women only

|  |  | **Germany** |  |  |  |  | **US** |  |  |  |
| --- | --- | --- | --- | --- | --- | --- | --- | --- | --- | --- |
| **Age** | **N** | **RS** | **SE** | **Plausibility range** |  | **N** | **RS** | **SE** | **Diff** | **P (Model)** |
| **15-24** | 108 | 59.5 | 6.2 | 56.3-59.5 |  | 176 | 69.3 | 5.1 | -9.8 | 0.1867 |
| **25-39** | 92 | 43.9 | 7.6 | 39.2-43.9 |  | 181 | 47.1 | 5.3 | -3.2 | 0.7864 |
| **40-59** | 134 | 37.2 | 5.7 | 33.7-37.2 |  | 294 | 24.9 | 4.0 | +12.3 | 0.4310 |
| **60-69** | 128 | 25.1 | 6.8 | 22.8-25.1 |  | 108 | 22.0 | 5.9 | +3.1 | 0.2618 |
| **Overall^a^** | 462 | 42.4 | 3.3 | 39.0-42.1 |  | 759 | 41.8 | 2.5 | +0.6 | 0.5813 |

N=number of cases

RS=5-year relative survival

SE=standard errors

Diff=difference in survival between Germany and the United States

a Age-standardized
